# Topping-off technique prevents aggravation of degeneration of adjacent segment fusion revealed by retrospective and finite element biomechanical analysis

**DOI:** 10.1186/s13018-014-0142-z

**Published:** 2015-01-28

**Authors:** Zhenqi Zhu, Chenjun Liu, Kaifeng Wang, Jian Zhou, Jiefu Wang, Yi Zhu, Haiying Liu

**Affiliations:** Division of Orthopaedics, Peking University People’s Hospital, Beijing, 100000 People’s Republic of China

**Keywords:** Spinal fusion, Topping-off operation, Biomechanics, Finite element analysis

## Abstract

**Aim:**

The aim of this study was to evaluate the effect of the Topping-off technique in preventing the aggravation of degeneration caused by adjacent segment fusion.

**Methods:**

Clinical parameters of patients who underwent L5-S1 posterior lumbar interbody fusion + interspinous process at L4-L5 (PLIF + ISP) with the Wallis system (Topping-off group) were compared retrospectively with those of patients who underwent solely PLIF. Pre- and post-operative x-ray measurements, visual analogue scale (VAS) scores, and Japanese Orthopaedic Association (JOA) scores were assessed in all subjects. Normal L1-S1 lumbosacral finite element models were established in accordance with the two types of surgery in our study, respectively. Virtual loading was added to assess the motility, disc pressure, and facet joint stress of L4-L5.

**Results:**

There were 22 and 23 valid cases included in the Topping-off and PLIF groups. No degeneration was observed in either group. Both VAS and JOA scores improved significantly post-operatively (*P* < 0.01). The intervertebral angle and lumbar lordosis of L4-L5 were both significantly increased (*t* = −2.89 and −2.68, *P* < 0.05 in the Topping-off group and *t* = −2.25 and −2.15, *P* < 0.05 in the PLIF group). In the Topping-off group, x-ray in dynamic position showed no significant difference in the angulation or distance of the anterior movement of the L4-L5 segment. The angle of hyper-extension and distance of the posterior movement of L4 were significantly decreased. In the PLIF group, both hyper-flexion and hyper-extension and posterior movement were increased significantly. In finite element analysis, displacement of the L4 vertebral body, pressure of the annulus fibrosus and nucleus pulposus, and stress of the bilateral facet joint were less in the Topping-off group under loads of anterior flexion and posterior extension. Facet joint stress on the left side of the L4-L5 segment was also less in the Topping-off group under left flexion loads.

**Conclusion:**

Short-term efficacy and safety between Topping-off and PLIF were similar, whilst the Topping-off technique could restrict the hyper-extension movement of adjacent segments, prevent back and forth movement of proximal vertebrae, and decrease loads of intervertebral disc and facet joints.

## Introduction

Posterior lumbar intervertebral fusion (PLIF) is the conventional treatment for lumbar degeneration entailing however a series of complications, amid which acceleration of adjacent segment degeneration (ASD) remains the major problem. Some scholars suggest that prophylaxis application of an interspinous process (ISP) or spacer in fusion segments could decelerate this process [1,2]. The Topping-off technique is a newly developed surgical technique, which combines rigid fusion with an interspinous process device in the adjacent segment to prevent ASD. Nonetheless, there are few reports on Topping-off surgery and its rationality, and the indications remain unclear. Finite element analysis was first introduced in spine biomechanics in the 1970s with a wide application ever since, as the methodology is minimally impacted by other parameters [3]. In the current study, we have compared the outcomes, safety, motility, and interspinous space between patients who underwent Topping-off and conventional PLIF techniques. Also, we used a finite element model to assess the motility, disc pressure, and facet joint stress under virtual loads *in silico*. We therefore aim to reveal the role of the Topping-off technique in preventing the aggravation of ASD.

## Materials and methods

### General information

Patients between Jan 2008 and Mar 2010 diagnosed with lumbar disc herniation or lumbar spinal stenosis in our centre were included. Informed consents were obtained from all patients, and the study was approved by the local ethical committee (Peking University People’s Hospital Institutional Review Board). The Topping-off technique was suggested to patients with mild L4-L5 disc degeneration (e.g. MODIC1) not meeting the criteria for internal fixation fusion as a supplement to conventional PLIF. Choice of surgery was at patient’s discretion. The Topping-off group consisted of 22 patients who underwent L5-S1 PLIF + L4-L5 ISP (Wallis), among which there were 14 men and 8 women with an average age of 44.5 years (21 to 64 years). The PLIF group consisted of 23 patients who underwent L5-S1 PLIF solely, among which there were 11 men and 12 women with an average age of 40 years (12 to 77 years). Pre-operative standing lumbar anteroposterior and lateral, flexion, and hyper-extension x-ray examinations were performed. All patients were evaluated pre-operatively for visual analogue scale (VAS) and Japanese Orthopaedic Association (JOA) scores.

### Device used and surgical technique

The Wallis system was provided by Abbott Spine (Bordeaux, France). The device has notches that fit the physiological shape of the spinous processes. It also has a flat band for best spread of constraints in contact with the bone to ensure the rigidity of the unstable segment, whilst preserving its mobility. The design and materials minimize the need for bony resection and avoid any concentration of constraint on the bone. Pedicle screws (XIA) were provided by Stryker Spine (Cestas, France). The cross-connectors were provided by Stryker Spine (XIA) and Moss Miami (DePuy Spine, Sarl, Switzerland). Fusion cages were provided by Stryker PEEK, Synthes, and Plivios. Choice of internal fixation was according to intraoperative calibrations. Posterior L5-S1 partial laminectomy, decompression discectomy, implantation of the PEEK fusion device, and bilateral L5-S1 pedicle fixation were performed in all patients. Autologous bone grafting was applied in the surgeries, in which too much bony structures were resected and stability could be compromised. Patients in the Topping-off group were additionally implanted with Wallis at L4-L5 as the interspinous spacer fixation after L5-S1 decompression fusion. All surgeries were performed by the same surgeon. Patients were bedded for 1 week post-operatively and were discharged with lumbar supports.

### Follow-ups

Patients were followed at post-operative months 1, 3, 6, and 12 and annually thereafter with standing lumbar anteroposterior and lateral, flexion, and hyper-extension x-ray examinations. VAS and JOA evaluations were also performed at the last follow-up.

### Radiological measurement

All measurements were conducted by a blinded orthopaedist using the GE Healthcare Centricity RIS CE V2.0 software. The anterior height (aDH) and posterior height (pDH) of the L4-L5 disc space, sagittal diameter of the L4 vertebral body (*W*), L4-L5 intervertebral angle (α), and lordosis of the upper endplates of L1-S1 (β) were measured on lateral x-ray images. Flexion (θ^+^) and hyper-extension angles (θ^−^) of the L4-L5 intervertebral space and the anterior and posterior slipping distance of the posterior edge of L4 (AO and RO, respectively) were measured on flexion and hyper-extension x-ray images. All length parameters were expressed in relative values to avoid magnification bias.

### Criteria for adjacent segment degeneration

Degeneration was defined radiologically as follows: 1) percentage of anterior and posterior lumbar slipping of >25% [4], 2) dynamic angulation of the interspinous space of >10° [5], 3) complete loss of the interspinous space [6], and 4) recurrence and aggravation of back pain post-operatively with no positive radiological findings and with exclusion of surgery-related complications [2].

### Statistical analysis

Data were presented as mean ± standard deviation and were processed with SPSS ver. 21.0 software. The two-tailed Student’s *t*-test was used for comparisons between the two groups. A *P* value of <0.05 was accepted as statistically significant.

### Finite element model

**Figure 1:**
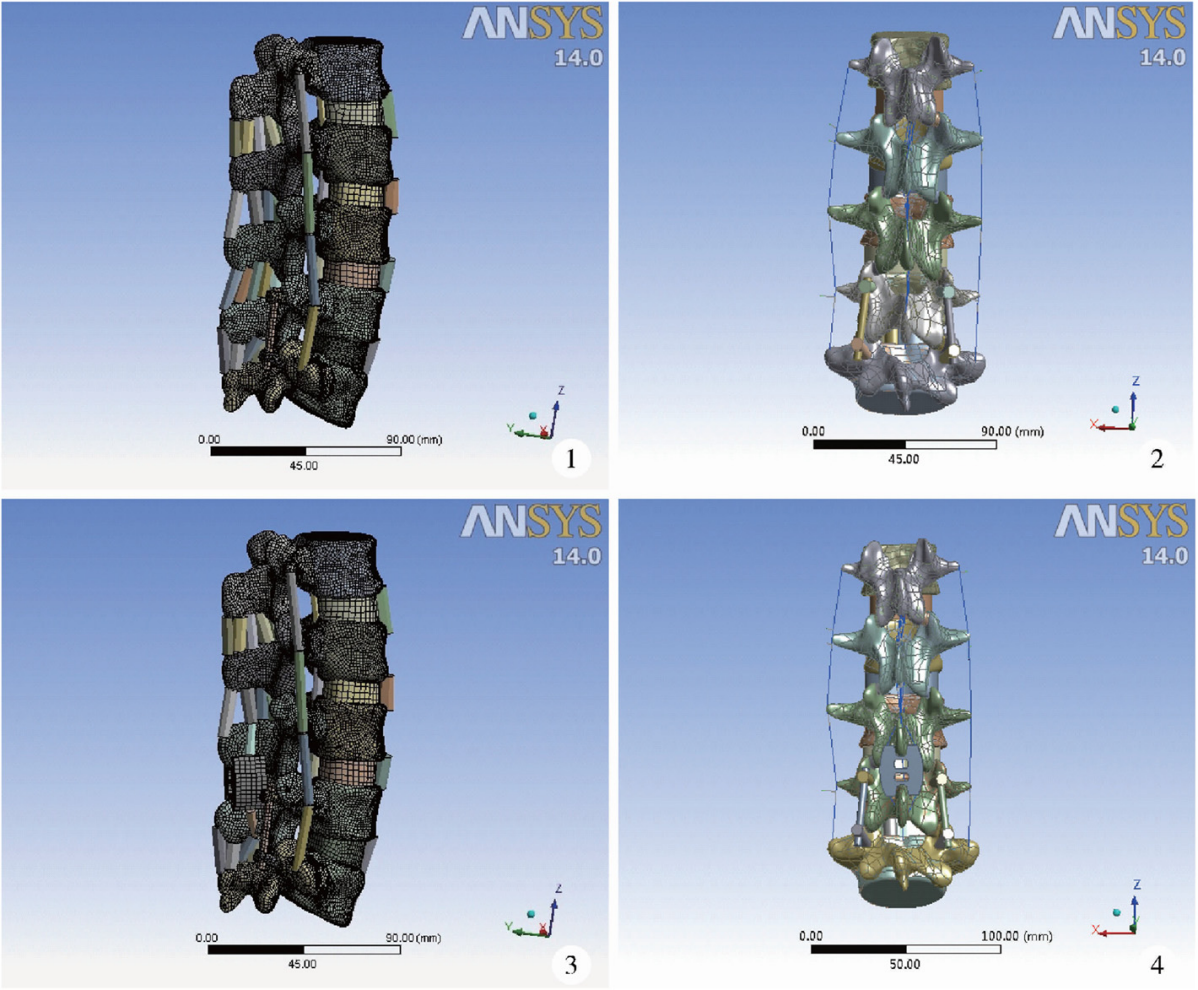
**Lateral and posterior aspects of the PLIF and Topping-off models.** (1) Lateral aspect of PLIF. (2) Posterior aspect of PLIF. (3) Lateral aspect of Topping-off. (4) Posterior aspect of Topping-off.

Serial thin-section CT images of L1-S1 of a healthy male volunteer were analysed using Mimics, Geomagic, and Ansys. A three-dimensional finite element model of L1-S1, which consisted of 459,340 units and 661,938 nodes, was constructed. After setting the boundary condition, unit type, and dividing finite element mesh, the S1 vertebra was constrained and loading was added. Five hundred newtons of vertical compressive loading was added and distributed evenly to each node of the endplate surface of the L1 vertebra. Ten newton metres of flexion, extension, left flexion, and right rotation torque loading was added, and average rigidity at all directions was calculated. Results were validated by reviewing previous reports. After exclusion of the supraspinous ligament, interspinous ligament, yellow ligament, part of the spinous process, part of the lamina, part of the nucleus in the gap, and part of the annulus of L5/S1, pedicle screws, connecting rods, and a single cage were implanted to obtain the PLIF model (Figure 1 (1 ~ 2)). The Topping-off model was constructed based on the PLIF origin by further excluding the supraspinous ligament, interspinous ligament, and part of the spinous process of L4-L5 and by implanting the Wallis ISP system (Figure 1 (3 ~ 4)). Relative L4 displacement, pressure on the annulus and nucleus, and stress on the bilateral facet joints of both models were measured four times in flexion, extension, left flexion, and right rotation status, and the mean value was calculated. In order to compare our model to the others, we did a thorough literature search by means of PubMed/MEDLINE under the search term “finite element” in combination with all keywords relevant to our subject of interest. Only full text published in English or Chinese were selected. Studies that covered all our parameters were eventually selected.

## Results

Twenty-two patients in the Topping-off group were followed for a mean duration of 24.8 months (12–40 months). Twenty-three patients in the PLIF group were followed with a mean duration of 23.7 months (12–38 months). There was no statistical significance in follow-up time (*P* > 0.05).

Pre-operative VAS score of 7.6 ± 1.2 dropped to 1.9 ± 0.9 post-operatively in the Topping-off group, and the difference reached statistical significance (*t* = 26.8, *P* < 0.01). Pre-operative VAS score of 6.8 ± 1.2 dropped to 1.9 ± 0.9 post-operatively in the PLIF group, and the difference also reached statistical significance (*t* = 26.9, *P* < 0.01). Better improvement in VAS score was noted in the Topping-off group over the PLIF group (*P* < 0.01). Pre-operative JOA score of 12.7 ± 2.9 improved to 22.5 ± 3.5 post-operatively in the Topping-off group, and the difference reached statistical significance (*t* = −14.6, *P* < 0.01). Pre-operative JOA score of 13.7 ± 1.9 improved to 23.2 ± 2.8 post-operatively in the PLIF group, and the difference also reached statistical significance (*t* = −16.9, *P* < 0.01). The improvement of JOA score did not differ significantly between the two groups. No patient presented symptoms indicative of ASD or implant rupture or loosening.

Standing lateral x-ray showed that both anterior (aDH/*W*) and posterior (pDH/*W*) disc height of L4-L5 increased in both groups, but the difference did not reach statistical significance (*P* > 0.05). Compared with pre-operative status, both groups showed a significant change in α post-operatively (*t* = −2.89, *P* < 0.05 in the Topping-off group; *t* = −2.25, *P* < 0.05 in the PLIF group). Compared with pre-operative status, both groups also showed a significant increase in β post-operatively (*t* = −2.68, *P* < 0.05 in the Topping-off group; *t* = −2.15, *P* < 0.05 in the PLIF group). The parameters measured on lateral x-ray are summarized in Table 1.

**Table 1:** **Measurement of parameters in the two groups on lateral x-ray (mean ± standard deviation)**

**Group**	**Time point**	**aDH/W**	**pDH/W**	**α (°)**	**β (°)**
Topping-off	Pre-op	0.46 ± 0.05	0.22 ± 0.05	8 ± 3	33 ± 11
	Post-op	0.48 ± 0.06	0.20 ± 0.04	11 ± 4	42 ± 10
PLIF	Pre-op	0.46 ± 0.07	0.23 ± 0.04	10 ± 4	32 ± 12
	Post-op	0.49 ± 0.05	0.21 ± 0.04	13 ± 5	40 ± 15

Hyper-flexion and hyper-extension x-ray showed that at last follow-up, the θ^+^ angle of L4-L5 and anterior movement of the posterior edge of L4 were not significantly changed compared with pre-operative status in the Topping-off group (*P* > 0.05). The θ^−^ angle and posterior movement of the posterior edge of L4 were significantly decreased (*t* = 5.57 and 4.75, respectively, *P* < 0.01). The parameters measured on hyper-flexion and hyper-extension x-ray are summarized in Table 2. Compared with pre-operative status, the PLIF group showed a significant increase in motility angle post-operatively both at hyper-flexion and hyper-extension positions (*t* = −5.77, *P* < 0.05 and *t* = −5.01, *P* < 0.01, respectively). The PLIF group also showed a significant distance increase in both anterior and posterior L4 movements post-operatively (*t* = 6.69 and *t* = −24, respectively, *P* < 0.01).

**Table 2:** **Measurement of parameters in the two groups on hyper-flexion and hyper-extension x-ray (mean ± standard deviation)**

**Group**	**Time point**	**θ** ^**+**^ **(°)**	**θ** ^**−**^ **(°)**	**AO/** ***W***	**RO/** ***W***
Topping-off	Pre-op	1.8 ± 0.5	3.2 ± 1.0	5.7 ± 1.3	2.1 ± 0.7
	Post-op	1.6 ± 0.5	1.8 ± 0.6	6.1 ± 1.5	1.2 ± 0.4
PLIF	Pre-op	2.0 ± 0.7	3.5 ± 1.0	5.4 ± 1.2	1.2 ± 0.3
	Post-op	3.7 ± 1.2	5.3 ± 1.4	8.1 ± 1.5	5.4 ± 0.7

The rigidity parameters we acquired in our finite element model in comparison with other reports are summarized in Table 3. Upon flexion-extension loading, there was less relative displacement of L4 in the Topping-off group than in the PLIF group (*P* < 0.05). However, upon flexor and rotation loading, relative L4 displacement was not significantly different between the two groups (Table 4). Upon flexion-extension loading, there was less pressure on the annulus and nucleus and less stress on the bilateral facet joint of the L4-L5 section in the Topping-off group than in the PLIF group (*P* < 0.05), whilst the difference was no longer statistically significant upon flexor and rotation loading (Table 5). Upon left flexion, there was less stress on the left facet joint in the Topping-off group (*P* < 0.05), whilst the difference lost statistical significance upon other types of loading (Table 6).

**Table 3:** **Rigidity in the current study and other reports**

	**Torque (Nm)**	**Flexion (Nm/°)**	**Extension (Nm/°)**	**Lateral flexion (Nm/°)**	**Rotation (Nm/°)**
Our cohort (L1-L5)	10	1.18	2.47	1.49	3.46
Yamamoto et al. (L1-L5) [7]	10	1.75	3.22	2.44	5.26
Heth et al. (L2-S1) [8]	6	1.10	2.35	1.33	2.61
Zhang et al. (L3-L5) [9]	10	1.62	3.03	2.50	4.45
Dong et al. [10]	10	2.35	3.58	2.86	8.98

**Table 4:** **Relative L4 displacement (mm)**

	**PLIF**	**Topping-off**	***t***	***P***
Flexion	1.4543 ± 0.1978	1.0928 ± 0.6694	3.462	0.013*
Extension	0.2616 ± 0.1569	0.1814 ± 0.2913	4.847	0.003*
(Left) flexion	0.8894 ± 0.6686	0.9548 ± 0.1002	1.087	0.319
(Right) rotation	1.4004 ± 0.5401	1.3447 ± 0.0084	2.037	0.088

*P < 0.05.

**Table 5:** **Pressure of L4-L5 annulus and nucleus (MPa)**

	**Annulus pressure**		**Nucleus pressure**	
	**PLIF**	**Topping-off**	**PLIF**	**Topping-off**
Flexion	0.8034 ± 0.0263	0.5823 ± 0.0945*	0.1753 ± 0.0475	0.0949 ± 0.0429*
Extension	0.2112 ± 0.0135	0.1882 ± 0.0040*	0.5654 ± 0.0328	0.4993 ± 0.0177*
(Left) flexion	0.4819 ± 0.4431	0.4302 ± 0.0439	0.5686 ± 0.0095	0.5787 ± 0.0115
(Right) rotation	0.4819 ± 0.4431	0.4491 ± 0.0173	0.1359 ± 0.0167	0.1446 ± 0.0317

*P < 0.05.

**Table 6:** **Left and right facet joint pressure of the L4/L5 segment**

	**Left facet joint pressure**		**Right facet joint pressure**	
	**PLIF**	**Topping**-**off**	**PLIF**	**Topping**-**off**
Flexion	6.535 ± 0.3719	3.793 ± 0.5276*	5.513 ± 0.1506	4.106 ± 0.2261*
Extension	5.2268 ± 0.2783	3.6318 ± 0.4171*	5.4821 ± 0.1339	3.7186 ± 0.2831*
(Left) flexion	6.5005 ± 0.1256	6.1333 ± 0.2222*	3.606 ± 0.2373	3.6972 ± 0.3444
(Right) rotation	4.0364 ± 0.0755	4.0501 ± 0.0879	5.8094 ± 0.2181	5.7974 ± 0.3311

*P < 0.05.

## Discussion

Degeneration of the interspinous disc is thought to be a physiological process, and spine fusion is believed to accelerate the process of ASD. The incidence of ASD was 5.2% to 18.5% based on symptoms and was up to 100% according to radiological evaluation [5]. Current risk factors for acceleration of degeneration included preoperative degenerative presentation, long segment fusion, low fusion, and sagittal imbalance. Upper adjacent segments were more prone to degenerate than lower ones, due to the compensatory increase of post-fusion adjacent motility and increased loading onto the disc and facet joints [5,11].

ISP systems, like Wallis or Coflex, can constrain lumbar hyper-flexion and hyper-extension, decrease pressure on the disc and facet joints, and thereby at the mean time reserve part of motility in the corresponding segments [12,13]. The Topping-off technique is therefore introduced by implanting ISP in the adjacent segments for protection. Nonetheless, prophylaxis ISP implantation using the Topping-off technique remains disputable. In a prospective cohort study, Korovessis et al. suggest that the Topping-off technique can alter the natural disease course of ASD and thereby reduce the incidence of the disease [2]. We suggest that the Topping-off technique has the following advantages [1]: 1) Topping-off can avoid fusion of segments with pre-existing degeneration and minimize fusion length, 2) Topping-off can protect the adjacent segments in the case of long segmental fusion and can offer transition to the unaffected segments, and 3) implantation of ISP can lower the complexity for potential second surgery.

Virginie et al. have combined a carcass Wallis surgery model with finite element biomechanics and have revealed that Wallis, as an ISP, can reduce the lumbar flexion-extension motility and disc pressure of adjacent segments, but may increase stress of the corresponding spinal processes [14]. There have been a number of retrospective and carcass studies investigating ISP devices, yet there has been no report integrating a retrospective study with finite element analysis. Finite element analysis works as a virtual biomechanical tool and warrants mechanical and clinical validation.

In the current study, the retrospective analysis covered symptomology and radiology and evaluated the trend of post-operational alteration of the adjacent segments. The finite element model, on the other hand, demonstrated the cumulative change of the adjacent segmental motility and stress of adjacent segments. Both modalities showed similar trends of alteration over time. The finite element model also revealed the change of stress of the adjacent segment and in part interpreted the outcomes from the retrospective analysis in a biomechanical perspective. In the retrospective part, the short-term outcome and safety between Topping-off and PLIF were similar, as revealed by post-operative indices and radiologic measurements. No degeneration of the adjacent segment was seen in any patient. The flexion-extension x-ray image revealed that ISP implantation restricted the segment extension and kept the anterior displacement of the adjacent segment similar to pre-operative status. Whereas the PLIF group did not meet the radiologic criteria for ASD, the motility of the adjacent segment and L4 slipping distance were both increased. As the Topping-off technique could prevent relative displacement, it could potentially reduce the risk of degeneration of the implanted segments. In the finite element analysis, less relative displacement of L4 vertebrae was noticed in the Topping-off group under flexion-extension loading, indicating that Topping-off not only reserved part of the motility of the adjacent segment but also restricted the extension of the spine via intraspinous cushion and restricted the flexion via ligation, thus increasing the stability of the adjacent segment. There was also less pressure of the annulus and nucleus and less facet joint stress upon L3/L4 in the Topping-off group under flexion-extension loading. This indicates that Topping-off can change the stress conduction of the spine during flexion and extension, and ISP and the spinal process share the loadings, in particular the loading onto the disc and facet. Of note, our study has provided a convincing advantage of implicating the Topping-off technique in addition to conventional PLIF, as the outcome in terms of VAS score in our Topping-off cohort is statistically better than that in PLIF. Although not adjusted or confirmed in a larger population, this implies a potential advantage of the novel technique over the conventional one in the selected patient group. These two techniques, rather than in the dispute of replacing one another, may cater for different subgroups of patients who were once lacking multiple modalities.

Nonetheless, our study has limitations. First, the retrospective nature of this study brings about inevitable bias, which warrants trials in the future to validate the results. Second, the sample size in the current study is still limited with relatively short follow-up time. The long-term outcome of the Topping-off technique is still awaited. Third, our study solely aimed at a single segment with ISP implantation, and study on long segment fusion with ISP is warranted. Though the finite element model of the L1-S1 segment has been established in the current study, more integral models of the lumbar region are needed for the study of lumbar biomechanics. Like most finite element studies, we did not simulate the muscular tissue. This could lead to deviation of the results. Also, due to the inherent limitation of the Wallis system, the effect of Topping-off may differ from clinical outcomes. Our results therefore are limited to profile the outcome of Wallis as an ISP.
